# Task-related activity in sensorimotor cortex in Parkinson's disease and essential tremor: changes in beta and gamma bands

**DOI:** 10.3389/fnhum.2015.00512

**Published:** 2015-09-22

**Authors:** Nathan C. Rowland, Coralie De Hemptinne, Nicole C. Swann, Salman Qasim, Svjetlana Miocinovic, Jill L. Ostrem, Robert T. Knight, Philip A. Starr

**Affiliations:** ^1^Department of Neurological Surgery, University of California, San FranciscoSan Francisco, CA, USA; ^2^Department of Neurology, University of California, San FranciscoSan Francisco, CA, USA; ^3^Helen Wills Neuroscience Institute, University of California, BerkeleyBerkeley, CA, USA; ^4^Department of Psychology, University of California, BerkeleyBerkeley, CA, USA

**Keywords:** Parkinson's disease, essential tremor, deep brain stimulation, electrocorticography, oscillations, ipad, beta frequency

## Abstract

In Parkinson's disease patients in the OFF medication state, basal ganglia local field potentials exhibit changes in beta and gamma oscillations that correlate with reduced voluntary movement, manifested as rigidity and akinesia. However, magnetoencephalography and low-resolution electrocorticography (ECoG) studies in Parkinson's patients suggest that changes in sensorimotor cortical oscillations differ from those of the basal ganglia. To more clearly define the role of sensorimotor cortex oscillatory activity in Parkinson's, we performed intraoperative, high-resolution (4 mm spacing) ECoG recordings in 10 Parkinson's patients (2 females, ages 47–72) undergoing deep brain stimulation (DBS) lead placement in the awake, OFF medication state. We analyzed ECoG potentials during a computer-controlled reaching task designed to separate movement preparation from movement execution and compared findings to similar invasive recordings in eight patients with essential tremor (3 females, ages 59–78), a condition not associated with rigidity or akinesia. We show that (1) cortical beta spectral power at rest does not differ between Parkinson's and essential tremor patients (*p* = 0.85), (2) early motor preparation in Parkinson's patients in the OFF medication state is associated with a larger beta desynchronization compared to patients with essential tremor (*p* = 0.0061), and (3) cortical broadband gamma power is elevated in Parkinson's patients compared to essential tremor patients during both rest and task recordings (*p* = 0.004). Our findings suggest an oscillatory profile in sensorimotor cortex of Parkinson's patients that, in contrast to the basal ganglia, may act to *promote* movement to oppose the anti-kinetic bias of the dopamine-depleted state.

## Introduction

Recent theories describing the network dynamics of abnormal movement patterns, such as rigidity and akinesia, in Parkinson's disease emphasize the role of pathological oscillatory synchronization of distributed neuronal populations. In local field potential recordings of subthalamic nucleus in Parkinson's patients, beta desynchronization during performance of motor and cognitive tasks has been shown to be attenuated in the OFF medication state compared to the ON medication state (Oswal et al., 2012). Additionally, broadband high gamma activity in motor cortex is associated with movement generation (Crone et al., 1998a) and this frequency range is also elevated in PD motor cortex compared to other disorders (Crowell et al., 2012). Taken together, these frequency band changes in the OFF medication state have been suggested as a possible basis for the symptoms of bradykinesia and bradyphrenia, that is, reduced motor and cognitive flexibility in Parkinson's patients (Engel and Fries, 2010; Jenkinson et al., 2013). However, it is not clear whether these changes are specific to basal ganglia or occur in parallel in other parts of the basal ganglia-thalamocortical motor loop. An earlier study began to address this question by examining sensorimotor cortex in Parkinson's patients undergoing deep brain stimulation (DBS) lead placement using low-resolution (1 cm spacing) electrocorticography (ECoG) during a simple stop-move task. In comparison to patients with essential tremor, the study showed that Parkinson's patients in the OFF medication state have elevated cortical broadband gamma power during the stop phase of the task, however no major differences in beta power during the stop or move phases were observed (Crowell et al., 2012).

In this study, we analyze beta and gamma frequency band dynamics in sensorimotor cortex of rigid-akinetic Parkinson's patients in the OFF medication state using higher resolution cortical recordings than in previous work (Crowell et al., 2012) that allow more precise electrophysiologic isolation of primary motor vs. primary sensory responses. In addition, we perform recordings during a computer-controlled task in which movement preparation and execution are dissociated. Data were collected intra-operatively in patients undergoing insertion of DBS leads into the basal ganglia and compared to similar recordings in patients without rigidity or akinesia undergoing DBS for essential tremor. All recordings were performed prior to penetration of deep structures (by microelectrodes or DBS leads) to avoid the confound of the acute “microlesion” effect (Koop et al., 2006). We show that both movement-related beta desynchronization and broadband gamma power in sensorimotor cortex in Parkinson's patients in the OFF medication state are enhanced during task performance in comparison with a non-parkinsonian disorder, suggesting a compensatory effort by the parkinsonian cortex to sustain motor activation in the setting of basal ganglia disease.

## Materials and methods

### Subjects

All subjects were patients who had consented to undergo implantation of deep brain stimulators for symptomatic treatment of Parkinson's disease or essential tremor. Inclusion criteria were: a diagnosis by a movement disorders neurologist of idiopathic Parkinson's disease with prominent rigid-akinetic features and motor fluctuations, or a diagnosis of essential tremor with action tremor inadequately responsive to therapy, in the setting of optimal medication management.

Preoperative characterization of the severity of motor signs was performed using the Unified Parkinson's Disease Rating Scale for Parkinson's disease and the Fahn-Tolosa-Marin Tremor Rating Scale for essential tremor (Fahn et al., 1987, 1993). Parkinson's patients were withdrawn from their antiparkinsonian medications for at least 12 h prior to surgery. Informed consent for the study was obtained in accordance with the Declaration of Helsinki using a protocol approved by the UCSF Committee on Human Research (IRB no. 10-01350). The consent process included an explicit discussion of the temporary introduction of a cortical electrode for research purposes only.

### ECoG strip insertion

We used a custom-designed 70 × 10 mm subdural electrode strip containing 28 contacts (AdTech, Racine, WI) positioned in a parasagittal plane over sensorimotor cortex. The contacts were spaced at 4 mm intervals center-to-center in 2 rows of 14 contacts each. Each circular, flat contact measured 2 mm in diameter and was exposed by a 1.2 mm diameter opening of the silastic covering. For bilateral cases, the strip was positioned on the side with clearest anatomic demarcation of the central sulcus (all patients), or, for Parkinson's disease, contralateral to the side of the most severe motor symptoms. The strip was inserted posteriorly through the same 15 mm burr hole used subsequently for DBS lead insertion. The burr hole was typically placed 4–6 cm anterior to the central sulcus, 3–4 cm from midline. Once inserted into the burr hole, a lateral x-ray image was taken to confirm placement of the strip over precentral gyrus as judged by the strip's position relative to a radioopaque marker placed on the scalp (Crowell et al., 2012). The ECoG strip was removed prior to securing the DBS lead, immediately after performing an intraoperative CT scan for documentation of ECoG electrode location (described below).

### Behavioral conditions

**Figure 1:**
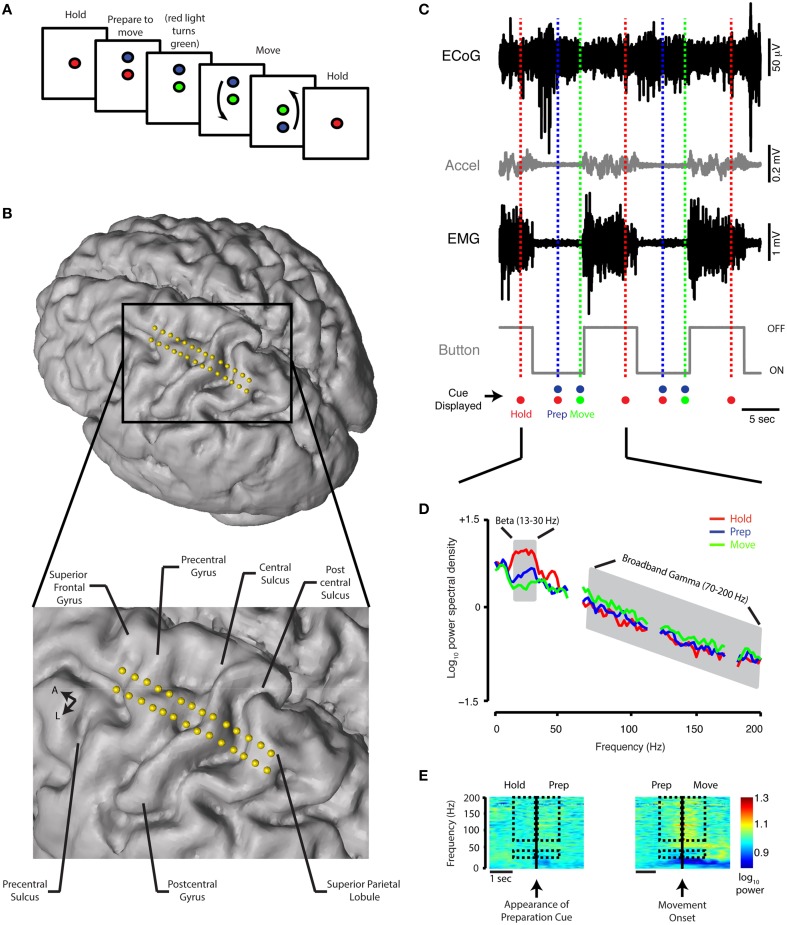
**Behavioral task, contact localization, examples of raw data recording, and spectral analysis. (A)** Schematic of the reaching task. See Materials and Methods for detailed description. **(B)** Electrode positions relative to the brain anatomy of a single subject. The cortical surface was reconstructed offline using the preoperative MRI (Statistical Parametric Mapping 8, SPM8). Electrode coordinates (in relation to the midpoint of the line adjoining the anterior and posterior commissures) were then determined by co-registering the preoperative MRI and intraoperative CT scan and mapped onto the cortical surface. **(C)** A 30-s simultaneous recording of ECoG data (from precentral gyrus), accelerometer position, EMG potential, and button tracing from a single Parkinson's subject. The time scale beneath the button recording indicates 5 s. The recordings are shown in relation to the timing of the HOLD, PREPARATION, and MOVE periods during two trials. Accel, accelerometer; m, milli; sec, second; μ, micro; V, volt. **(D)** Power spectral density for the HOLD, PREPARATION and MOVE periods are superimposed [average of 20 trials, same subject as **(C)**. The PSD curve for the REST period for this subject is nearly indistinguishable from the HOLD PSD curve and is not shown. Gray shaded areas indicate boundaries of the beta (13–30 Hz) and broadband gamma (70–200 Hz) frequency bands. **(E)** Spectrograms for task transitions, same ECoG data as depicted in **(C)**. Hashed marks indicate the frequency bands (beta: 13–30 Hz and broadband gamma: 70–200 Hz) and intervals (1 s before and after the appearance of the blue filled circle and movement onset for the HOLD-PREP and PREP-MOVE transitions, respectively) over which spectrogram data are shown for comparison.

ECoG data were collected with patients quietly resting with their eyes open for 1–2 min (denoted as REST period) in addition to a movement task consisting of a hold period (HOLD), a movement preparation period (PREPARATION) and a movement execution period (MOVE) (schematic in Figure 1A). The task was displayed on a 16-gigabyte iPad device (Apple Inc., Cupertino, CA) secured into a customized flat screen holder facing the patient and attached to the surgical bed. Prior to initiating task recordings, patients practiced several trials of the task. The initial display of the task consisted of a filled red circle present in the center of the screen for 5 s (HOLD). This was followed by the appearance of a filled blue circle either above or below the red dot (PREPARATION). After 3–5 s with both circles on the screen, the red circle became green in color, indicating the MOVE period, during which the patient lifted their forearm from a button resting in their lap and touched the screen at the location of the blue circle. After successful contact with the screen at this location, the blue circle continued to cycle above and below the green circle 6–8 times. The patient was instructed to touch the blue dot at each new location while it was cycling. To end the trial, the red circle again appeared alone on the screen, prompting the patient to return their forearm to the resting position. The number of presentations of the blue dot was chosen so that the duration of the MOVE period would be similar to the duration of the other periods. During a single presentation of the dot in the MOVE period, if no touch was detected after 2–5 s (adjusted individually for the subject based on movement speed), the blue circle would cycle automatically to the other position. The interval between two consecutive appearances of the red circle constituted a single trial. The diameter of the blue circle was adjusted between 1 and 3 cm prior to the beginning of the recording based on patient visual acuity and line of sight. Patients completed up to 20 trials per session.

### Data recording

All recordings occurred prior to any brain penetration with microelectrodes, guide tubes, or the DBS lead. All ECoG channels were recorded in monopolar configuration, with a needle in the scalp serving as ground and reference, and sampled at 3051.8 Hz (System 3, Tucker-Davis Technologies, Alachua, FLA.).

The upper limb contralateral to the cortical recording was monitored with a tri-axial accelerometer wrist band (AX2300-365, FHC, Inc., Bowdoin, ME) as well as surface EMG electrode pads (Bagnoli-2 EMG system, Delsys, Inc. Natick, MA) over the flexor carpi radialis and biceps muscles. (In one patient, accelerometry was not available, and in two separate patients, EMG of flexor carpi radialis was not available.) When not moving, patients were instructed to rest their hand on a custom-built button placed on their lap (a 9 × 11.5 × 11.75 cm square platform, the top of which was outfitted with a button).

Analog signals from the EMG electrodes, accelerometer, and button were routed through separate preamplifiers prior to connection with the analog-to-digital converter (sample rate = 24,414.1 Hz). The appearance of the red circle on the iPad screen was cosynchronous with a 200 ms internally generated tone relayed to the analog-to-digital converter.

### Data analysis

Data were analyzed offline using MATLAB R2014a (MathWorks, Natick, MA).

#### Pre-processing

All signals were digitally downsampled to 1 kHz. Notch filters were applied at 60 Hz and harmonics to eliminate power line noise artifacts. For each recording, the average signal derived from all ECoG channels without excessive noise was subtracted from each separate ECoG channel (common average referencing).

#### Spectral analysis

A 10- to 30-s continuous epoch of activity during the REST period was identified after removing episodes of noise artifacts in the 1–2 min recording. Within the extracted REST segment, any epochs of ECoG activity coinciding with spontaneous movements as judged by simultaneous deflections in both the accelerometer and EMG traces were further excluded (final average length of REST segment for all subjects = 24.67 ± 7.5 s, mean ± std, range = 10–30 s). From these 10- to 30-s segments, a 2-s epoch of REST activity was eventually chosen for final analysis as described below.

For the movement task, time-series recordings were synchronized by matching the timestamp indicating the beginning of the first trial and the first voltage deflection from the sound output within the analog recording of the iPad. Within individual trials, times of movement onset were manually identified using periods of increased EMG and/or accelerometry activity relative to baseline in addition to button tracings (accelerometer and button tracings closely matched EMG periods of increased activity and return to baseline—Figure 1C). Based on this visual analysis and the timestamps provided by the task software, individual trials were partitioned into HOLD (period between appearance of red circle and appearance of blue circle), PREPARATION (period between appearance of blue circle and transition of red to green circle), and MOVE (period between visually identified movement onset and offset) periods of activity. The length of the MOVE period varied between individual trials, however on average it was not different between groups (Parkinson's disease: 6.7 ± 1.2 s, range = 4.8–9.1 s; essential tremor: 7.6 ± 1.5 s, range = 5.2–9.9 s, mean ± std, *p*_rs_ = 0.21). Individual trials were discarded if movement occurred prior to the “move” cue (for all patients, median number of trials per patient = 13.5, range = 5–20).

We primarily focused our analysis on two frequency bands: beta frequency (13–30 Hz) and broadband gamma frequency (70–200 Hz). Asynchronous neuronal spiking is believed to be reflected in this very broad gamma band and thus serves as a surrogate for local cortical activation (Manning et al., 2009; Miller et al., 2009; see Supplementary figures for comparison with narrow gamma band analysis). To standardize the analysis of oscillatory activity across periods, 2-s epochs were extracted from the REST segment described above and each task period. These were used for calculation of power spectral density (PSD): the final 2 s of the HOLD period, the 2 s in the middle of the PREPARATION period and the first 2 s following movement onset within the MOVE period. In addition, to focus on the transitions between task periods, PSD was computed using 1-s epochs extracted before and after the occurrence of the preparation cue and movement onset for the HOLD to PREP transition and PREP to MOVE transition, respectively (see rectangles in Figure 1E).

PSD was computed using Welch's method (window = 512 samples, Hamming type; overlap = 50%) (Welch, 1967). To compare differences between periods (i.e., REST, HOLD, PREPARATION, or MOVE), PSD estimates were computed for the REST epoch and, for the task periods, averaged over the number of included trials and base 10 log-transformed (hereafter referred to as “log,” Figure 1D). To compare changes in PSD at the transitions, a difference in log power during consecutive 1-s epochs was calculated.

Spectrograms (Figures 1E, **3A**) were computed for display using the short-time Fourier transform (window = 512 samples, Hamming type; overlap = 90%) (Allen, 1977) aligned on preparation cue (for HOLD to PREP transition) and time of movement onset (for PREP to MOVE transition). For averaging across subjects, spectrogram data were transformed into a modified z-score in which the median of the spectrogram values for each frequency band over a 4-s epoch was computed and compared to the last second of “HOLD” as baseline (**Figure 3A**).

#### Contact localization

All subjects underwent preoperative brain MRI and CT for stereotactic planning prior to frame-based DBS electrode implantation. After implanting the DBS lead, an intraoperative CT scan (Medtronic O-arm) was obtained to confirm superposition of the DBS lead with its planned trajectory and to determine ECoG electrode contact coordinates.

ECoG electrode coordinates were reconstructed offline by co-registering the preoperative MRI and intraoperative CT scans (STEALTH FrameLink® software, Medtronic, Minneapolis, MN). The central sulcus was identified by noting the appearance of a posteriorly oriented “Omega-sign” indicating the location of the “hand knob” in the precentral gyrus (Yousry et al., 1997). Other identification criteria included orthogonal orientation of the superior frontal sulcus to the precentral sulcus and reversal of the somatosensory evoked potential (SSEP) N20 potential marking the postcentral gyrus (Crowell et al., 2012). Based on these criteria, individual electrode contacts were assigned to the following anatomic locations: postcentral sulcus (PoCeSu), postcentral gyrus (PoCeGy), central sulcus (CenSul), precentral gyrus (PrCeGy), and precentral sulcus (PrCeSu) (Figure 1B). Additionally, 12 out of 18 subjects and 11 out of 18 subjects had coverage over superior frontal gyrus (SuFrGy) and superior parietal lobule (SuPaLo), respectively (Table 1). PSD data from contacts covering a single area were averaged for multiple subjects prior to further statistical analysis.

**Table 1:** **Number of contacts per cortical region in Parkinson's and essential tremor patients**.

**Region**	**Parkinson's disease**		**Essential tremor**		*p*_rs_^*^
	**Total no. of contacts**	**Median contacts/sbj**	**Total no. of contacts**	**Median contacts/sbj**	
SuFrGy	46	5	30	4	0.81
PrCeSu	24	2	20	2	1.00
PrCeGy	64	6	48	6	0.81
CenSul	34	2	24	2	1.00
PoCeGy	48	4	38	6	0.66
PoCeSu	34	3	22	2	0.89
SuPaLo	30	2	42	5	0.20

*CenSul, Central sulcus; no., number; PoCeGy, postcentral gyrus; PoCeSu, postcentral sulcus; PrCeGy, precentral gyrus; PrCeSu, precentral sulcus; sbj, subject; SuFrGy, superior frontal gyrus; and SuPaLo, superior parietal lobule*.

* *p-value resulting from comparison of average number of contacts per cortical area between the Parkinson's and essential tremor groups (using rank sum test). No comparisons reached statistical significance*.

#### Statistical analysis

One-sample Kolmogorov-Smirnov tests (Arnold and Emerson, 2011) revealed data sets used for most comparisons did not meet criteria for normal distribution, thus all statistical analyses were performed using non-parametric testing (Wilcoxon rank-sum test for comparing 2 groups and Kruskal-Wallis test for comparing 3 or more groups). *P*-values resulting from two-group statistical tests were corrected using the false discovery rate method (Storey, 2002). Tests comparing 3 or more groups were first screened for corrected *p* values less than 0.05 (using false discovery rate). Those tests meeting this criterion underwent *post-hoc* analysis to discover significant intergroup differences, the *p*-values of which were corrected using the Bonferroni method, which are the final corrected values reported. For analyzing repeated measures across periods in individual subjects in each disease state, a one-factor Friedman test was used. Fisher's exact test was used to compute *p*-values from contingency tables. *P*-values are subscripted by test type (fet, Fisher's Exact test, 2-tailed; frt, Friedman's test; kw, Kruskal-Wallis test; rs, Wilcoxon rank-sum test).

## Results

### Subject characteristics

Ten Parkinson's patients (2 females, average age at surgery = 58.4 ± 9.3 years, mean ± std) and eight essential tremor patients (3 females, average age at surgery = 68.9 ± 6.7 years, mean ± std) were included in the current study. Demographics are provided in Tables 2, 3. Although the two groups did not differ with respect to gender distribution (*p*_fet_ = 0.61), there was a significant difference with respect to age (*p*_rs_ = 0.025). In Parkinson's disease, the average UPDRS tremor rating at rest (part III, item 20) in the upper extremity contralateral to the recorded hemisphere was 1.7 ± 1.5 (mean ± std) out of a possible total of 4 points. In essential tremor, the average tremor rating (Fahn-Tolosa-Marin Tremor Rating Scale, part A—rest, posture and action total) for the upper extremity contralateral to the recorded hemisphere was 5.8 ± 2.5 (mean ± std) out of a possible total of 12 points. For the Parkinson's group, subthalamic nucleus was the target for eight patients and globus pallidus for two. Six of ten Parkinson's patients had simultaneous bilateral implantations. Seven essential tremor patients underwent motor thalamic implantation, two of which were bilateral. One essential tremor patient underwent thalamotomy.

**Table 2:** **Parkinson's disease patient characteristics**.

**Pt**	**Age at surgery (years)**	**Gender**	**Duration of disease (years)**	**UPDRS III off medication**	**UPDRS III on medication**	UPDRS III tremor off^*^ medication
1	65	M	15	43	10	3
2	47	M	6	53	26	1
3	49	F	6	37	10	3
4	58	F	11	37	22	0
5	51	M	15	48	28	0
6	61	M	10	33	17	1
7	70	M	5	17	2	2
8	48	M	5	32	9	4
9	63	M	4	32	8	0
10	72	M	6	43	30	3

Duration of disease, interval between diagnosis and initial surgery; F, female; M, male; Pt, patient; UPDRS III, Unified Parkinson's Disease Rating Scale part III;

* *UPDRS resting tremor score (part III, item 20) for upper extremity contralateral to recorded hemisphere*.

**Table 3:** **Essential tremor patient characteristics**.

**Pt**	**Age at surgery (years)**	**Gender**	**Duration of disease (years)**	**FTM**	FTM^*^
1	67	M	50	ND	ND
2	78	F	37	ND	ND
3	59	M	40	42	6
4	65	F	20	75	9
5	63	M	13	ND	ND
6	73	F	6	68	3
7	77	M	25	ND	ND
8	69	M	10	55	5

F, female; FTM, Fahn-Tolosa-Marin Tremor Rating Scale (total of parts A, B, and C); M, male; ND, not documented; Pt, patient;

* *FTM resting, postural and action tremor score (part A) for upper extremity contralateral to recorded hemisphere*.

### Signal amplitudes and overview of movement-related changes

Signal amplitudes were similar for both patient groups. Root-mean-square signal amplitudes over all cortical areas (after common average referencing for all task recordings) were 26.10 ± 10.5 μV for Parkinson's patients and 23.55 ± 9.6 μV for essential tremor patients (mean ± std, *p*_rs_ = 0.22). For contacts overlying precentral gyrus, root-mean-square signal amplitudes were 38.0 ± 10.3 μV and 32.5 ± 15.2 μV for Parkinson's and essential tremor patients, respectively (*p*_rs_ = 0.17).

Consistent with prior work on movement-related sensorimotor cortex ECoG potentials (Crone et al., 1998a,b; Miller et al., 2007), the onset of movement was generally associated with a beta decrease and gamma increase (individual subject example in Figure 1E). Both of these spectral changes began prior to movement onset. In both groups, the MOVE period was associated with a significant decrease in beta power and increase in gamma power when log power was averaged over all cortical areas (*p* < 0.001).

### Cortical beta power does not differ between disease groups

**Figure 2:**
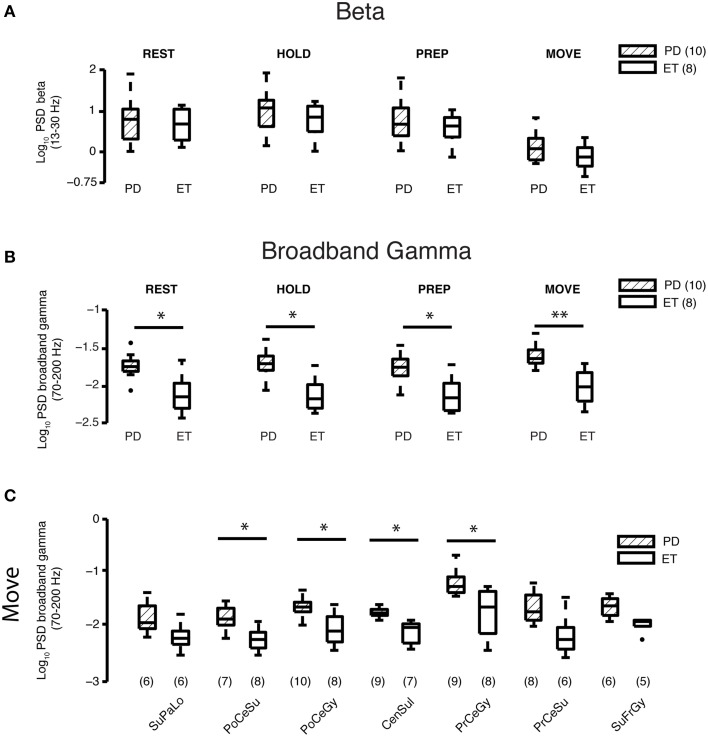
**Beta and gamma spectral power at REST and during different task periods. (A)** Comparison of average log_10_ beta power between Parkinson's disease (*n* = 10 subjects) and essential tremor (*n* = 8 subjects) groups for the REST and task periods. Horizontal line within the boxplot indicates median value, lower and upper box limits indicate the interquartile range (25 and 75%, respectively) and whiskers extend to minimum and maximum values of the dataset. Filled dark circles indicate outliers. No differences were found for any period. ET, essential tremor; PD, Parkinson's disease. **(B)** Comparison of average log_10_ broadband gamma power between Parkinson's disease (*n* = 10 subjects) and essential tremor (*n* = 8 subjects) groups for the REST and task periods. Boxplots constructed as in **(A)**. ^*^p_rs_ < 0.05, ^**^p_rs_ < 0.01, refer to Results section for specific *p*-values. ET, essential tremor; PD, Parkinson's disease. **(C)** Cortical topography of the broadband gamma frequency band differences between Parkinson's disease and essential tremor groups, illustrated here for the MOVE period. Boxplots constructed as in **(A)**. Values in parentheses indicate number of patients with contact coverage of a particular cortical region. CenSul, central sulcus; ET, essential tremor; PD, Parkinson's disease; PoCeGy, postcentral gyrus; PoCeSu, postcentral sulcus; PrCeGy, precentral gyrus; PrCeSu, precentral sulcus; SuFrGy, superior frontal gyrus; SuPaLo, Superior parietal lobule; ^*^p_rs_ < 0.05, refer to Results section for specific *p*-values.

Log beta power averaged over all cortical areas was similar in both groups for all periods (REST—Parkinson's disease: 0.78 ± 0.17, essential tremor: 0.66 ± 0.15, *p*_rs_ = 0.85; HOLD—Parkinson's disease: 0.99 ± 0.16, essential tremor: 0.77 ± 0.15, *p*_rs_ = 0.56; PREPARATION—Parkinson's disease: 0.78 ± 0.17, essential tremor: 0.62 ± 0.14, *p*_rs_ = 0.85 and MOVE—Parkinson's disease: 0.18 ± 0.11, essential tremor: −0.090±0.11, *p*_rs_ = 0.32, mean ± se, all units = log_10_(μV)^2^/Hz) (Figure 2A).

### Elevated broadband gamma power at REST and all task periods in parkinson's disease vs. essential tremor patients

Log broadband gamma power averaged over all cortical areas during the REST epochs and all task periods (HOLD, PREPARATION, and MOVE) was higher in the Parkinson's than essential tremor group (REST—Parkinson's disease: −1.75±0.05, essential tremor: −2.12±0.09, *p*_rs_ = 0.018; HOLD—Parkinson's disease: −1.72±0.06, essential tremor: −2.13±0.08, *p*_rs_ = 0.011; PREPARATION—Parkinson's disease: −1.75±0.06, essential tremor: −2.12±0.08, *p*_rs_ = 0.013; MOVE—Parkinson's disease: −1.60±0.05, essential tremor: −2.01±0.08, *p*_rs_ = 0.0038, mean ± SE, all units = log_10_(μV)^2^/Hz, Figure 2B). Individual cortical areas showing a significant difference in average log broadband gamma power between the Parkinson's and essential tremor groups during the MOVE period included postcentral sulcus (Parkinson's disease: −1.83±0.07, essential tremor: −2.24±0.07, *p*_rs_ = 0.013), postcentral gyrus (Parkinson's disease: −1.64±0.06, essential tremor: −2.04±0.1, *p*_rs_ = 0.034), central sulcus (Parkinson's disease: −1.73±0.03, essential tremor: −2.11±0.07, *p*_rs_ = 0.0027) and precentral gyrus (Parkinson's disease: −1.18±0.08, essential tremor: −1.73±0.16, *p*_rs_ = 0.019, Figure 2C). Precentral gyrus also showed a significant difference in average log broadband gamma power between the Parkinson's and essential tremor groups in the REST (*p*_rs_ = 0.0071), HOLD (*p*_rs_ = 0.0040), and PREP (*p*_rs_ = 0.010) periods (data not shown). In the Parkinson's group, average log broadband gamma power in contacts overlying precentral gyrus was highest among all other cortical areas during REST (*p*_kw_ = 5.0 × 10^−4^) and all task periods (HOLD *p*_kw_ = 4.6 × 10^−4^, PREP *p*_kw_ = 4.1 × 10^−4^, MOVE *p*_kw_ = 0.0012, Supplementary Figure 1). This result was further tested in which contacts overlying precentral gyrus and postcentral gyrus were directly compared using a 2-factor, 2-level ANOVA design. For the moving period, results showed significant gamma power elevation in PD vs. ET (*p* = 0.0001, main effects) and M1 vs. S1 (*p* = 0.0006, main effects) with a non-significant interaction term (*p* = 0.454, data not shown). In the essential tremor group, average log broadband gamma power was not different when compared across cortical areas during REST or any task period (data not shown).

While sensorimotor cortex gamma band responses to movement tend to occur in the “broadband” spectral distribution of 50–200 Hz (Crone et al., 1998a; Crowell et al., 2012), previous work on basal ganglia local field potentials in Parkinson's patients show levodopa-related changes in a more narrow spectral band of 60–90 Hz (Brown et al., 2001). Therefore, we sought to determine whether the disease-related differences in cortical gamma band power in the present study were driven primarily by changes in this narrower band. The gamma band was subdivided into the 60–90 Hz and 100–200 Hz ranges for additional analyses and showed, as with broadband gamma, elevation during REST and all task periods (Supplementary Figure 2) when the bands were analyzed independently.

Given that broadband gamma power levels distinguished Parkinson's from essential tremor both at rest and during task performance, we calculated the Pearson correlations between arm motor cortex gamma power and the severity of Parkinson's signs (UPDRS part III scores, off medication). We did not find a linear correlation (*r* = 0.09, *p* = 0.80 for resting state, Supplementary Figure 3), likely because all Parkinson's patients who present for surgical therapy are already in a moderately advanced state, without a great deal of between subject variability in severity (Table 2). These results were the same when the UPDRS part III scores were limited to contralateral upper extremity rigidity and bradykinesia subscores (item 22 + item 33, *p* > 0.05, data not shown).

### Beta decrease during early movement preparation is more pronounced in Parkinson's disease than essential tremor

**Figure 3:**
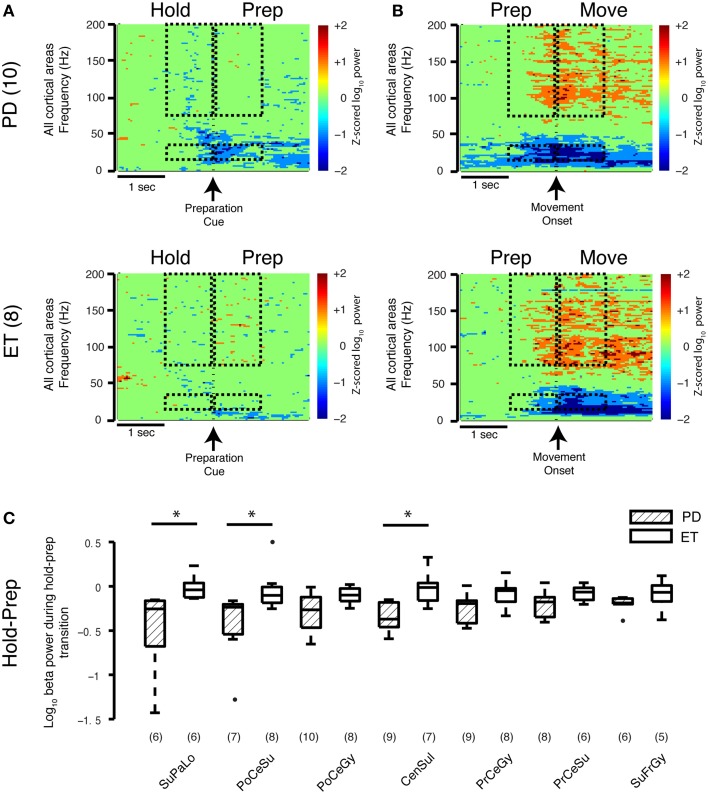
**Movement-related transitions in beta and gamma power**. **(A,B)** Average spectrograms during the HOLD-PREP and PREP-MOVE transitions for the Parkinson's disease (*n* = 10 subjects) and essential tremor (*n* = 8 subjects) groups. Hashed marks as in Figure 1E. Note that no calculations were performed on the spectrogram data. **(C)** Cortical topography of the beta band decrease during the early HOLD-PREP transition (i.e., comparison of difference in log PSD values in both groups). Values in parentheses indicate number of patients with contact coverage of a particular cortical region. Boxplot construction is the same as in Figure 2A. CenSul, central sulcus; ET, essential tremor; PD, Parkinson's disease; PoCeGy, postcentral gyrus; PoCeSu, postcentral sulcus; PrCeGy, precentral gyrus; PrCeSu, precentral sulcus; SuFrGy, superior frontal gyrus; SuPaLo, Superior parietal lobule, ^*^p_rs_ < 0.05, refer to Results section for specific *p*-values.

To study the transition between task periods, cortical beta power (all regions) was averaged 1 s before and 1 s after the appearance of the PREPARATION cue (HOLD-PREP transition) and movement onset (PREP-MOVE) for both groups. In the early movement PREPARATION period (HOLD-PREP transition), the average log beta power difference between the HOLD and PREPARATION periods decreased to a greater extent in the Parkinson's disease (−0.31±0.06) than essential tremor group (−0.071±0.05) (mean ± SE, all units = log_10_(μV)^2^/Hz, *p*_rs_ = 0.0061) (spectrogram data only shown in Figure 3A). In examining this transition in specific cortical locations, the stronger early preparation-related beta decrease was found in contacts overlying central sulcus (Parkinson's disease −0.34±0.05, essential tremor −0.031±0.07, *p*_rs_ = 0.022), postcentral sulcus (Parkinson's disease −0.44±0.13, essential tremor −0.043±0.08, *p*_rs_ = 0.032), and superior parietal lobule (Parkinson's disease −0.49±0.16, essential tremor −0.012±0.05, *p*_rs_ = 0.014) (Figure 3C). The average decrease in log beta power during the latter segment of the PREPARATION period was not significantly different between groups, nor were there disease specific differences in beta power at the prep-move transition (spectrogram data only shown in Figure 3B). Thus, the low-dopamine parkinsonian state is associated with a stronger cortical beta decrease during early movement preparation, compared with a non-parkinsonian condition.

Comparing Parkinson's to essential tremor, no disease-specific differences were found in the increase in broadband gamma power during either the HOLD-PREP (*p*_rs_ = 0.24) or PREP-MOVE transitions (*p*_rs_ = 0.46, see also average spectrograms shown in Figures 3A,B), and this was true for all cortical areas studied (data not shown). In summary, sensorimotor cortex broadband gamma power is elevated in all periods of the task in Parkinson's disease compared to essential tremor, however task-related *changes* in gamma power did not differ between Parkinson's disease and essential tremor.

## Discussion

In this study, we analyzed cortical oscillations in ECoG potentials recorded from subjects diagnosed with rigid-akinetic Parkinson's disease or essential tremor during DBS surgery. Parkinson's patients were studied only in the OFF medication state. Subjects were recorded during rest and during performance of a motor task that distinguished movement preparation from execution. While resting state beta power in sensorimotor cortex did not differ between Parkinson's disease and essential tremor, the task-related beta decrease associated with early movement preparation was more pronounced in Parkinson's disease than essential tremor. Additionally, we showed that power in the broadband gamma frequency band is higher in Parkinson's patients than essential tremor patients during rest—in agreement with Crowell et al. (2012)—and during all task periods, while task-related gamma changes did not distinguish the two groups. We found that these gamma band differences were most prominent in precentral gyrus encompassing primary motor cortex. These cortical findings differ from published findings for subthalamic nucleus local field potentials that report diminished movement-related beta changes and reduced gamma power in Parkinson's patients in the OFF medication state (Androulidakis et al., 2007; Oswal et al., 2012).

### Role of cortical beta desynchronization during movement preparation

In healthy individuals, sensorimotor cortical beta oscillations decrease in anticipation of an upcoming movement and remain desynchronized during the movement itself (Crone et al., 1998b; Miller et al., 2007; de Lange et al., 2008). These movement-related changes in beta oscillations are also present in basal ganglia local field potentials recorded from DBS electrodes in implanted Parkinson's patients. However, during the OFF medication state (associated with severe parkinsonian motor signs) movement-related beta decreases in basal ganglia are attenuated relative to the ON medication state (associated with less severe motor dysfunction) (Jenkinson and Brown, 2011; Oswal et al., 2012), lending support to the hypothesis that movement-related beta changes in basal ganglia are causally linked with motor performance in Parkinson's patients (Hammond et al., 2007; Little and Brown, 2014). Given the strong functional connection between motor cortex and subthalamic nucleus (Sharott et al., 2005; Lalo et al., 2008; Shimamoto et al., 2013), one might predict an impairment of movement-related beta decrease in cortex in Parkinson's patients OFF medication compared to non-parkinsonian conditions. However, we observed the opposite pattern.

With respect to network dynamics, the fundamental hallmark of the Parkinsonian state is the excessive synchronization of population spiking to a particular phase of the motor beta rhythm (Weinberger et al., 2006; Moran et al., 2008; de Hemptinne et al., 2013, 2015). This effect is proposed to reflect entrainment of population spiking in the Parkinson's disease motor system into a relatively inflexible pattern rendering the cortex less able to respond to sensory or cognitive signals for movement initiation (de Hemptinne et al., 2015). However, for movement to occur at all, the cortex must decouple population spiking from the beta rhythm in spite of this entrainment (Miller et al., 2012; Yanagisawa et al., 2012). Thus, one interpretation of the strong cortical beta decrease during movement preparation in Parkinson's disease is that it is a compensatory mechanism to counteract the exaggerated beta rhythm-spike field synchronization within the basal ganglia in Parkinson's disease. Hypothetically, akinesia could result when this cortical compensatory mechanism fails.

Resting and movement-related cortical beta changes in PD have also been studied by magnetoencephalography (MEG) (Stoffers et al., 2007; Pollok et al., 2009; Hirschmann et al., 2011; Litvak et al., 2011; te Woerd et al., 2014). While MEG studies generally agree with our result that resting cortical beta power does not differ between parkinsonian and non-parkinsonian conditions, one MEG study showed an impaired, rather than enhanced, movement-related beta decrease during movement preparation (te Woerd et al., 2014). The apparent conflict with our own study may be related to differences in the nature of the task, body part engaged, and method of cortical localization in MEG vs. ECoG studies.

### Elevated cortical broadband gamma power suggests increased population spiking

Cortical broadband activity is thought to be a surrogate measure of local population spiking and tracks at least some features of the positron emission tomography hemodynamic response (Shibasaki, 2008) and functional MRI blood oxygen level-dependent signal (Smith et al., 2002). Thus, increased cortical broadband activity implies an increase in cortical metabolic activity. Accordingly, Huang and colleagues, in a longitudinal study, demonstrated increasing fluorodeoxyglucose positron emission tomography metabolism in primary motor cortex with disease progression (Huang et al., 2007). Moreover, Ko and colleagues found that Parkinson's patients at rest had elevated uptake of ^15^O-water in primary motor cortex and cerebellum (but not basal ganglia) compared to healthy control subjects (Ko et al., 2013). This pattern worsened with disease progression.

Using low-resolution ECoG (1 contact/gyrus), we previously reported an increase in broadband gamma power at rest in Parkinson's patients relative to those with essential tremor and dystonia (Crowell et al., 2012). Here, we extend these earlier findings with high-resolution mapping of the cortical localization of this relative elevation in gamma power and, by using a motor task allowing us to isolate distinct movement periods, we show that this elevation persists during both movement preparation and execution. Our use of higher resolution ECoG array allowed us to examine signals associated with sub-regions of sensorimotor cortex whereas previous low resolution recordings may have sampled primary motor and primary sensory areas with a single contact, especially if the contacts spanned the central sulcus.

While the exact mechanisms by which motor cortex controls movement remain in dispute, it is likely that normal movement requires a minimum population of neurons to coordinate task-related activity (Shenoy et al., 2013). In the Parkinsonian state, we hypothesize that excessive spike-field synchronization holds a proportion of the neuronal pool in an active but rigid state that is unavailable for task-related functions. This active but effectively “useless” neuronal pool contributes to high levels of broadband activity during rest or during the HOLD period of a movement task, and it necessitates similarly elevated levels of neuronal activity during movement preparation and execution to achieve the necessary activation of available neuronal populations for movement initiation. Elevated broadband cortical gamma activity in Parkinson's disease OFF medications (compared to a non-parkinsonian disorder) is in contrast to the elevated subthalamic nucleus narrowband gamma activity observed in Parkinson's patients ON medication (compared to the OFF medication state) (Brown et al., 2001; Androulidakis et al., 2007). This may be because the specific frequencies of the gamma band that are relevant to parkinsonism may differ between cortex and basal ganglia, since our cortical findings appear to be true for “broadband gamma” (approximately 50–200 Hz), while subthalamic nucleus gamma frequencies relevant to parkinsonism are more narrowband in approximately 60–90 Hz (Brown et al., 2001; Androulidakis et al., 2007) or 250–300 Hz (López-Azcárate et al., 2010; Özkurt et al., 2011). Thus, any theoretical mechanism of compensation by the cortex may be independent of narrow band oscillations in STN and instead reflect local processing in cortex for which a more broadband gamma frequency range is the dominant rhythm. Moreover, it is unlikely that narrowband gamma signals (60–90 Hz) were driving the broadband gamma elevation in the PD group in this study, since similar changes were seen for gamma activity at 100–200 Hz.

### Limitations

Acute intraoperative recordings are constrained in the number of trials a patient can perform due to time limitations of the surgical procedure. Thus, we may miss more subtle changes (possibly within other spectral bands) that may be evident after averaging a greater number of trials. We were not able to study Parkinson's patients in the ON medication state due to surgical constraints. We did not sample the supplementary motor cortex due to the presence of bridging veins near the midline in most subjects. Because Parkinson's patients who undergo DBS implantation are in a moderately affected range, our Parkinson's disease cohort lacked sufficient range of pathological severity to correlate physiological findings with clinical disease severity. Our results could have been confounded by differences in movement kinematics between Parkinson's and essential tremor patients, but this possibility is mitigated by the fact that movement speed did not differ between the Parkinson's and essential tremor groups (see Materials and Methods), probably related to the instructions given to subjects to move deliberately rather than as fast as possible. Thus, group differences observed should not be due to a difference in actual motor performance. Finally, in performing invasive physiological studies in humans, normal controls are not ethically possible. Our approach to this was to use a comparison group of patients with a different disorder (essential tremor) but it is not possible to be certain which distinctions between groups represent an abnormality in one disorder vs. the other.

## Conclusion

We have shown that the movement-related cortical beta decrease is enhanced in Parkinson's patients OFF medication during a task involving a motor preparatory phase, compared to subjects with essential tremor. Broadband gamma activity, a surrogate for population spiking, is increased in all task phases in Parkinson's disease. These changes are distinct from those previously shown in basal ganglia local field potential recordings (Brown et al., 2001; Androulidakis et al., 2007). We hypothesize that the parkinsonian cortex may engage compensatory mechanisms to partially counteract the excessive spike-field synchronization induced by the dopamine-depleted state.

## Author contributions

NR, CD, NS, and PS were responsible for the conception of the manuscript, collection of data, data analysis and final revisions of the manuscript. SQ and SM participated in data collection and analysis. JO was responsible for clinical categorization and scoring of patient motor severity. RK was responsible for the conception and final revisions of the manuscript.

### Conflict of interest statement

The authors declare that the research was conducted in the absence of any commercial or financial relationships that could be construed as a potential conflict of interest.
