# Supplementary figures and images for: Task-related activity in sensorimotor cortex in Parkinson's disease and essential tremor: changes in beta and gamma bands
Source: Front Hum Neurosci. 2015 Sep 22;9:512. doi: 10.3389/fnhum.2015.00512 (PMC4585033; doi:10.3389/fnhum.2015.00512)

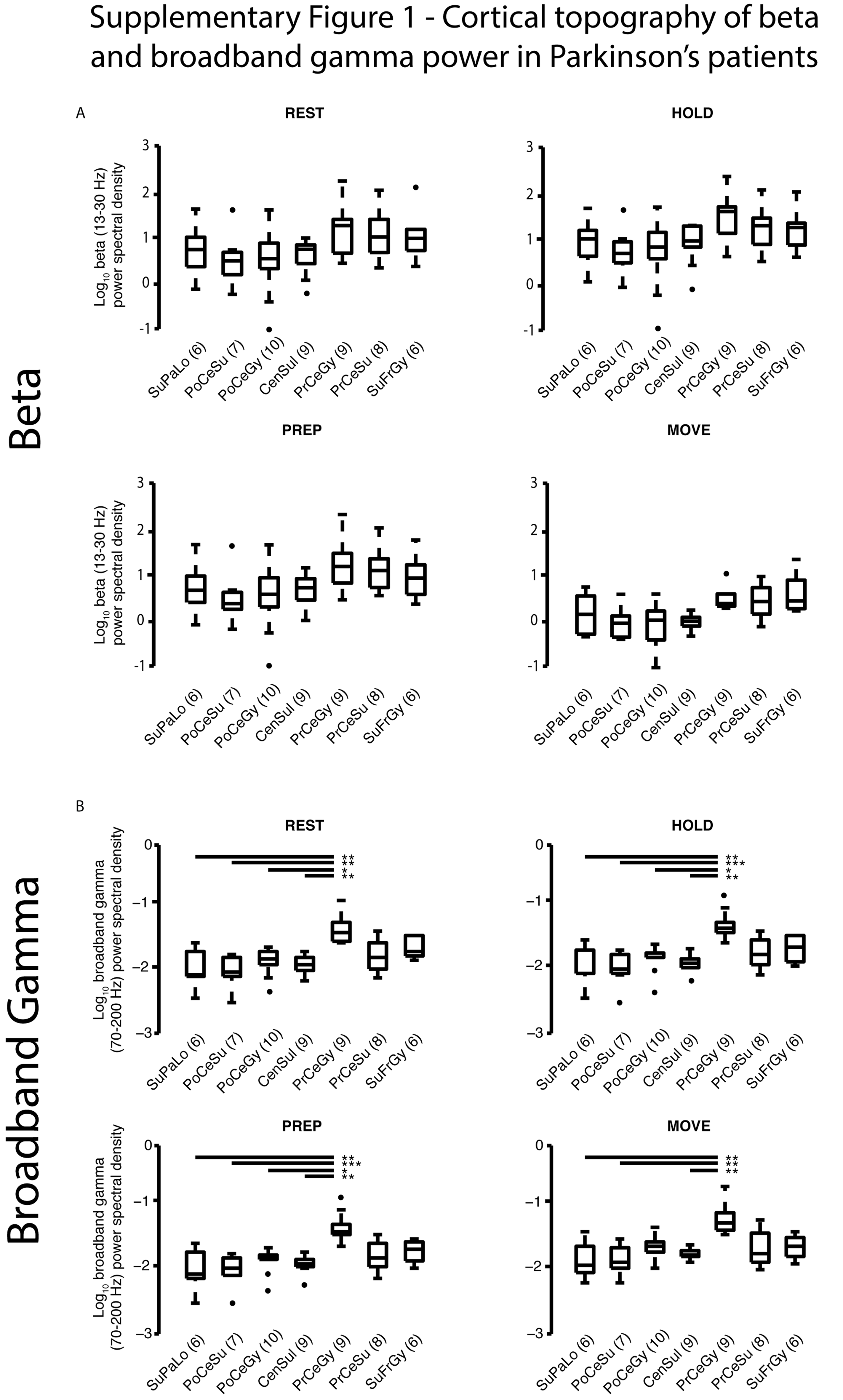

Supplement: Supplementary file 1 [file Image1.TIF]

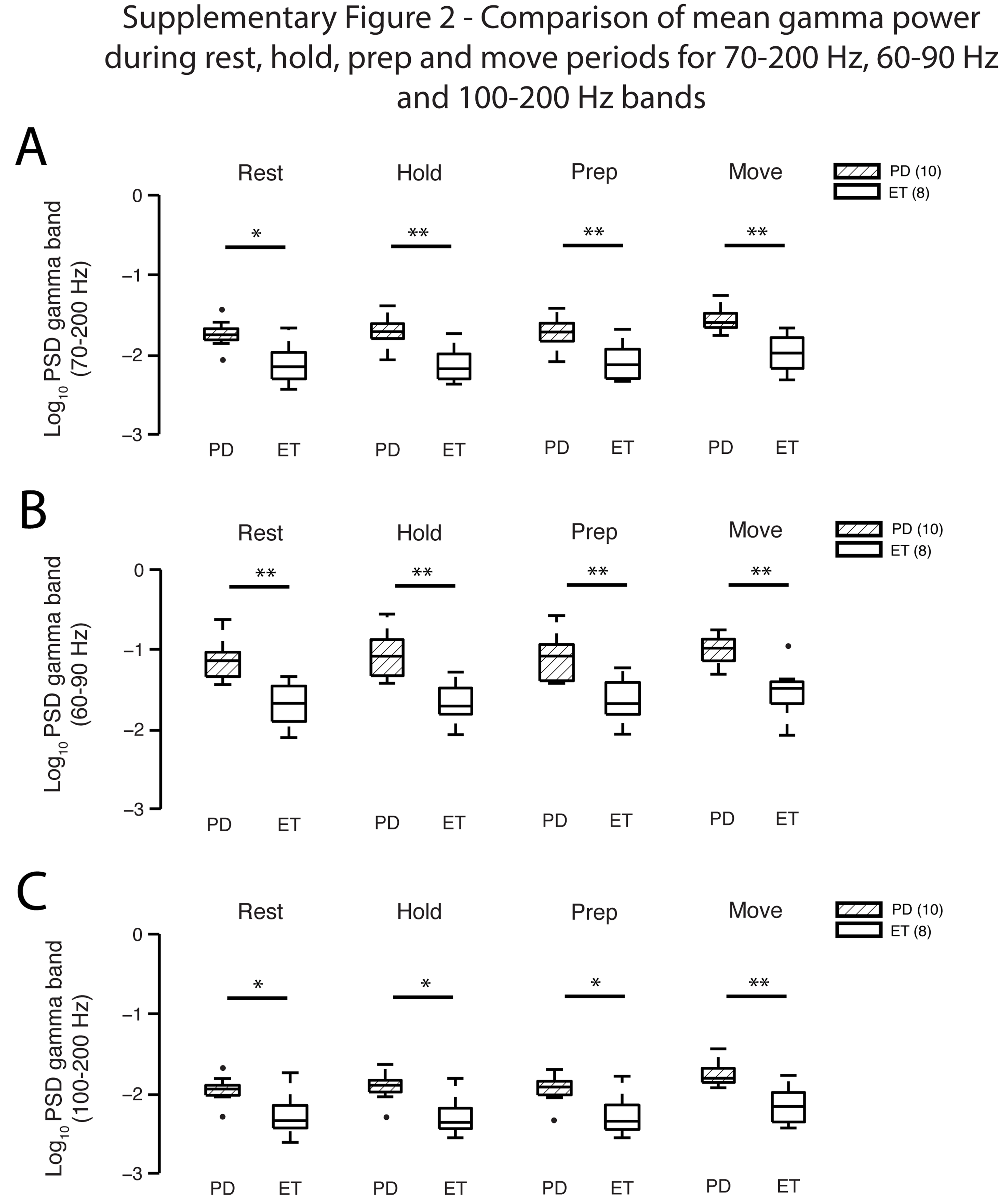

Supplement: Supplementary file 2 [file Image2.TIF]

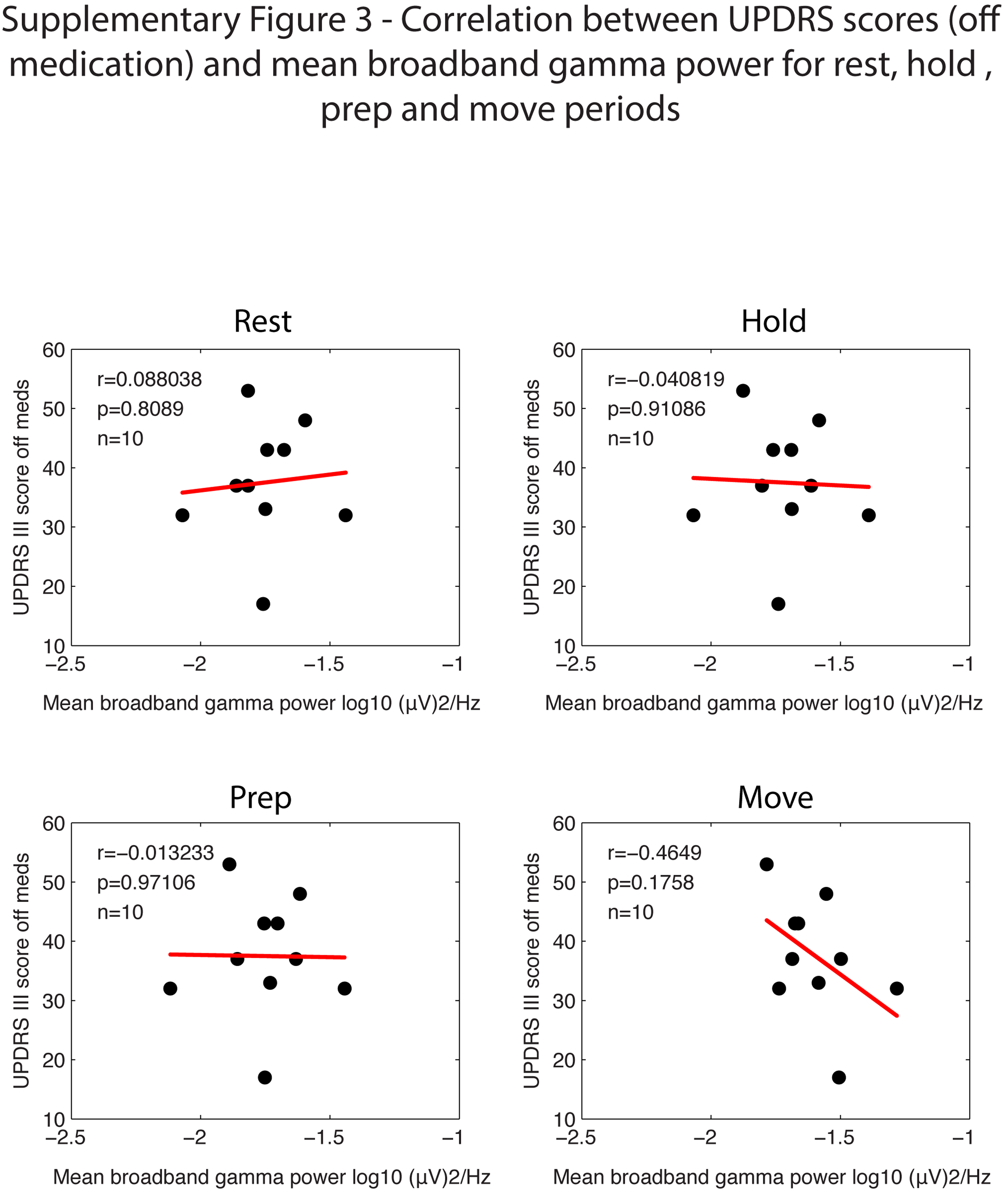

Supplement: Supplementary file 3 [file Image3.TIF]
